# Radioprotective Effect of Walnut Oligopeptides Against Gamma Radiation-Induced Splenocyte Apoptosis and Intestinal Injury in Mice

**DOI:** 10.3390/molecules24081582

**Published:** 2019-04-22

**Authors:** Na Zhu, Rui Liu, Li-Xia He, Rui-Xue Mao, Xin-Ran Liu, Ting Zhang, Yun-Tao Hao, Rui Fan, Mei-Hong Xu, Yong Li

**Affiliations:** 1 Department of Nutrition and Food Hygiene, School of Public Health, Peking University, Beijing 100191, China; summer920503@163.com (N.Z.); liuruipku@163.com (R.L.); helixiapku@163.com (L.-X.H.); maoruixue@163.com (R.-X.M.); liuhappy07@163.com (X.-R.L.); Zhangting930511@163.com (T.Z.); haoyuntaolly@163.com (Y.-T.H.); fanruirf@bjmu.edu.cn (R.F.); xumeihong@bjmu.edu.cn (M.-H.X.); 2 Department of Surgery, Beth Israel Deaconess Medical Center, Harvard Medical School, Boston, MA 02215, USA

**Keywords:** walnut oligopeptides, antioxidant, epithelial barrier, immunosuppression, splenocyte apoptosis

## Abstract

Walnut oligopeptides (WOPs) intake is associated with the augment of the antioxidant defense system and immune system. The chief object of this study is to evaluate the radioprotective effect of walnut oligopeptides extracted from walnut seed protein against ^60^Coγ-irradiation induced damage in mice. Female BALB/c mice were administered WOPs through drinking water for 14 days until a single dose of whole-body ^60^Coγ-irradiation. The 30-day survival test was carried out in the first group (8 Gy), and the other two groups (3.5 Gy) were sacrificed at 3 days and 14 days post-irradiation. Blood and organ samples of mice in the three groups were collected, the histopathological analysis and immunohistochemistry were conducted. The number of peripheral blood leukocytes, bone marrow DNA content, inflammatory cytokines, antioxidant capacity, and intestinal permeability were measured. We found that the administration of WOPs augmented antioxidant defense system, accelerated hematopoietic recovery and showed the significant trend toward higher survival rate and less weight loss compared with non-administrated control mice. In addition, WOPs administration appeared to be important to limit IR-induced splenocyte apoptosis and inflammatory cascade as well as reduce intestine epithelial barrier dysfunction and promote epithelial integrity. These results suggest that pre and post-treatment of WOPs may help to ameliorate acute damage, which is induced by ionizing radiation in mice and accelerate its recovery.

## 1. Introduction

Ionizing radiation (IR) produces a large amount of reactive oxygen species (ROS), which has harmful effects on cellular macromolecules like DNA, lipids, proteins, and inducing a series of biological consequences including cell apoptosis, inflammation, autoimmune reaction, ultimately causing impairment of several organs and systems and even death. Hematopoietic stem cells (HSCs) and hematopoiesis are among the tissues/organs most sensitive to radiation injury [1]. Hematopoietic failure caused by hematopoietic parenchymal cell injury and hematopoietic microenvironment damage also can be reflected in the changes of visible components in peripheral blood. The intestinal epithelium is the fastest renewing tissue in adult mammal [2], IR also attacks intestinal stem cells and results in damage to the intestinal epithelium. Thereby, disrupted intestinal barrier function and promoted the development of autoimmune and inflammatory diseases. Thus, reducing stress-induced barrier changes may have a therapeutic benefit [3,4,5]. Moreover, ROS also has a negative effect on antioxidant system, could induces the decreased level of glutathione (GSH) and the activity of antioxidant enzymes [6,7]. Therefore, as the exposure to IR, especially medical exposure, is regularly seen in our daily life, it becomes of great importance to seek for safe and effective radioprotectors.

There is a growing interest in the use of bioactive peptides as radioprotectors because of their high tissue affinity, specific and efficient biological activities including antioxidative activity, immunoregulatory activity, antimicrobial activity, etc. [8,9]. Raja et al. [10] found that fermented milk peptides had radioprotective potential, which demonstrated that milk peptides administration could lower cell deformation level and improve micronucleus formation in the albino mice and Catla catla fish blood cells. Our previous data suggested that ginseng oligopeptides exhibits effective therapeutic effects on attenuating irradiation-induced hematopoietic, gastrointestinal, and oxidative injury in mouse model by reducing systematic inflammatory and metabolic disorders [5]. While walnut (Juglans regia L) oligopeptides (WOPs) is extracted from walnut seed, which is very commonly consumed worldwide as organic snacks. WOPs have many useful properties including low molecular weight, high bioavailability and eases assimilation giving it numerous potential physiological functions. However, no available studies explored the radioprotective effect of WOPs. Therefore, the present study was performed to investigate the possible radioprotective effect of WOPs against the total body-radiation induced harmful effects in mice.

## 2. Results

### 2.1. Effect of WOPs on Survival Time after Whole-Body Irradiation

**Figure 1:**
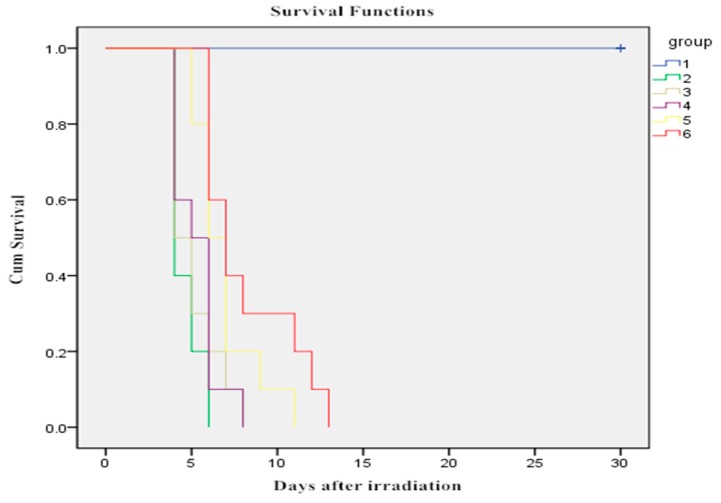
Effect of WOPs on survival time in mice after whole-body irradiation. Survival functions. 1: vehicle control group; 2: IR control group; 3: IR+whey protein group; 4: IR + WOPs 0.22 g/kg BW group; 5: IR + WOPs 0.44 g/kg BW group. 6: IR + WOPs 0.88 g/kg BW group.

Survival curves according to Kaplan-Meier showed: IR significantly reduced the survival time of IR control mice compared with those of vehicle control mice (*p* < 0.05). However, the intervention of WOPs at the dose of 0.44 and 0.88 g/kg BW exhibited the significantly prolonged survival time of irradiated mice (*p* < 0.05), when it was 6.90 ± 1.85 and 8.20 ± 2.74 days after irradiation (Figure 1). No deaths occurred in the vehicle control group.

### 2.2. Effects of WOPs on Body Weight and Immune Organ Indices in Mice after Whole-Body Irradiation 

**Figure 2:**
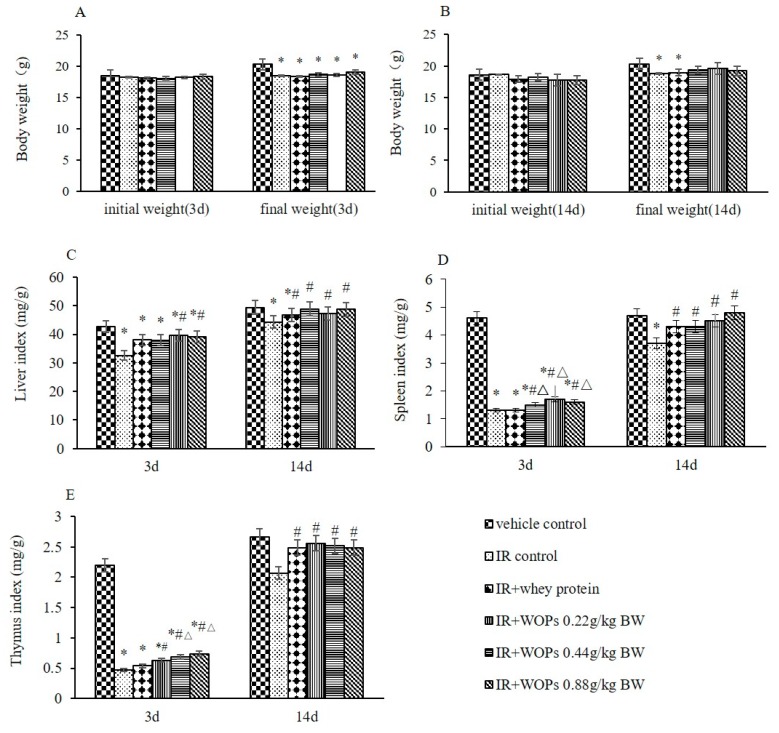
Effect of WOPs on body weight and immune organ indices in mice after whole-body irradiation. (**A**) Body weight 3d (g); (**B**) Body weight 3d (g); (**C**) Liver index (mg/g) = liver weight (mg)/ body weight (g); (**D**) Thymus index (mg/g) = thymus weight (mg)/ body weight (g); (**E**) Spleen index (mg/g) = spleen weight (mg)/ body weight (g). Values represented the mean ± S.D. (*n* = 10 per group), which were analyzed by ANOVA test followed by least significant difference for post hoc test between multiple groups. * *p* < 0.05 versus vehicle control group, # *p* < 0.05 versus IR control group, and Δ*p* < 0.05 versus IR + whey protein group.

Even though the significant reduction in the final weight of mice at 3 days postirradiation cannot be slowed down, WOPs significantly speeded up the weight back as the time of WOPs administration progressed (Figure 2A,B). Moreover, irradiation also resulted in the significant reduction in liver, spleen, and thymus index. WOPs significantly slowed down its decrease within 3 days after irradiation and the organ indexes have reached the vehicle control level at 14 days postirradiation (Figure 2C–E).

### 2.3. Effect of WOPs on White Blood Cell Count and Bone Marrow Hematopoietic System Damage

**Figure 3:**
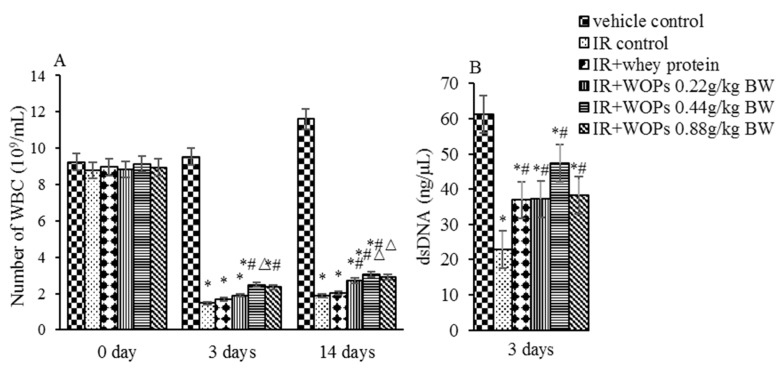
Effect of WOPs on white blood cell count and bone marrow hematopoietic system damage. (**A**) Number of WBC (10^9^/mL); (**B**) dsDNA (ng/μl). Values represented the mean ± S.D. (*n* = 10 per group), which were analyzed by ANOVA test followed by least significant difference for post hoc test between multiple groups. * *p* < 0.05 versus vehicle control group, # *p* < 0.05 versus IR control group, and Δ*p* < 0.05 versus IR + whey protein group.

Decreased white blood cell count and bone marrow DNA concentration in response to irradiation were observedat 3 days postirradiation compared with vehicle control mice, while the treatment with WOPs leads to considerable improvement (Figure 3). Moreover, WOPs administration exhibited significantly higher white blood cells count at 14 days after irradiation, demonstrating that WOPs administration has a better long-term effect than whey protein. 

### 2.4. Effect of WOPs on Irradiation-Induced Depletion of Endogenous Antioxidant Defense Systems

**Figure 4:**
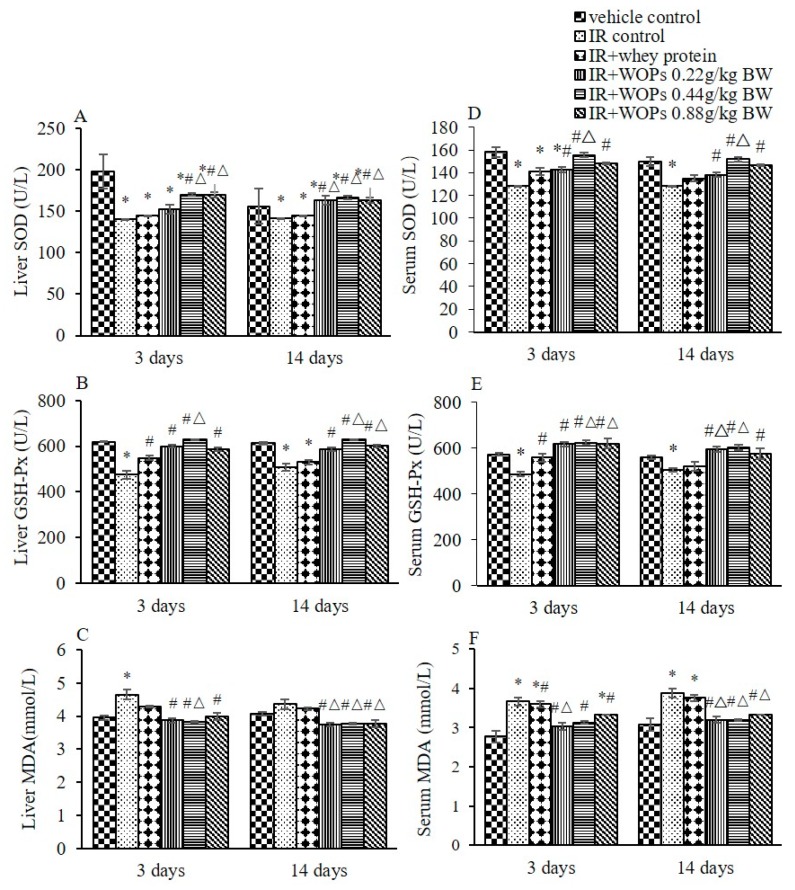
Effect of WOPs on irradiation-induced depletion of endogenous antioxidant defense systems. (**A**) Liver SOD (U/L); (**B**) Liver GSH-Px (U/L); (**C**) Liver MDA (mmol/L); (**D**) Serum SOD (U/L); (**E**) Serum GSH-Px (U/L); and (**F**) Serum MDA (mmol/L). Values represented the mean ± S.D. (*n* = 10 per group), which were analyzed by ANOVA test followed by least significant difference for post hoc test between multiple groups. * *p* < 0.05 versus vehicle control group, # *p* < 0.05 versus IR control group, and Δ*p* < 0.05 versus IR + whey protein group.

Irradiation significantly decreased the activities of SOD and GSH-Px in liver and serum, and induced a notable increase in MDA level at both 3 and 14 days postirradiation. A tendency towards higher SOD and GSH-Px activity as well as lower MDA level in liver and serum were seen in WOPs administration group at both 3 and 14 days postirradiation, which are more effective in long term than whey protein (Figure 4).

### 2.5. Effect of WOPs on Pro-Inflammatory Cytokine Levels in Serum

**Figure 5:**
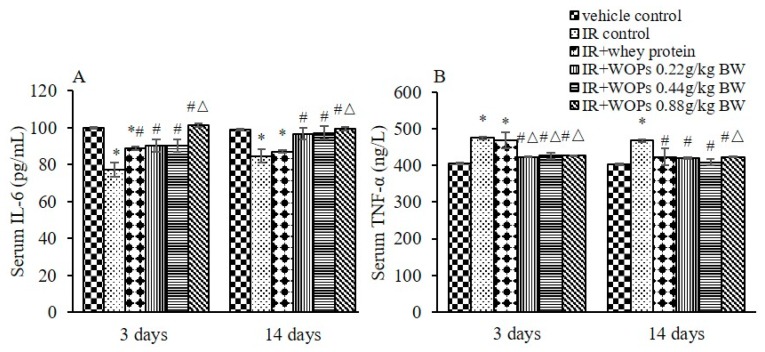
Effect of WOPs on pro-inflammatory cytokine levels in serum. (**A**) Serum IL-6 level; (**B**) Serum TNF-αlevel. Values represented the mean ± S.D. (*n* = 10 per group), which were analyzed by ANOVA test followed by least significant difference for post hoc test between multiple groups. * *p* < 0.05 versus vehicle control group, # *p* < 0.05 versus IR control group, and Δ*p* < 0.05 versus IR + whey protein group.

To evaluate the effect of WOPs on the content of pro-inflammatory cytokines in serum, the levels of IL-6 and TNF-α were measured. As shown in Figure 5A,B, serum IL-6 was decreased and TNF-α was increased by irradiation compared with vehicle control mice. Whereas the treatment with WOPs significantly increased the level of IL-6 and decreased the level of TNF-α in serum at both 3 and 14 days postirradiation. The effects of WOPs were better than whey protein, especially in WOPs 0.88 g/kg group. 

### 2.6. Effect of WOPs on Radiation-Induced Small Intestinal Mucosal Injury

**Figure 6:**
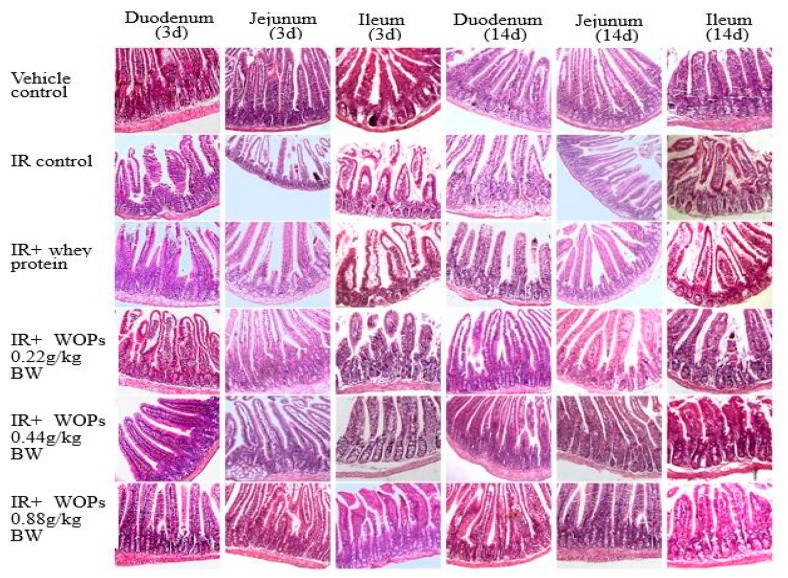
Effect of WOPs on intestinal morphology in irradiated mice (200×). Duodenum at 3 days, Jejunum at 3 days and Ileum at 3 days were intestinal morphology in mice at 3 days postirradiation; Duodenum at 14 days, Jejunum at 14 days and Ileum at 14 days were intestinal morphology in mice at 14 days postirradiation.

**Figure 7:**
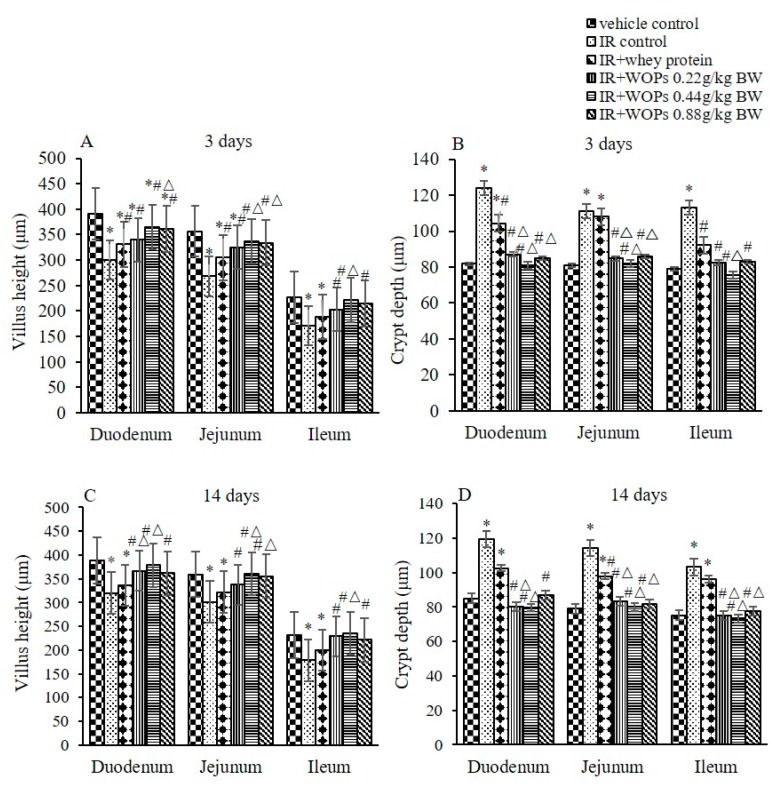
Effect of WOPs on villus height and crypt depth in irradiated mice. (**A**) Villus height at 3 days postirradiation; (**B**) Crypt depth at 3 days postirradiation; (**C**) Villus height at 3 days postirradiation; (**D**) Crypt depth at14 days postirradiation. Values represented the mean ± SD (*n* = 12 per group). * *p* < 0.05 versus vehicle control group, # *p* < 0.05 versus IR control, and Δ*p* < 0.05 versus IR+whey protein control group.

To determine whether intestinal mucosa was one of the targets for WOPs to exert its protective role, we examined the histology on 3 days and 14 days postirradiation. Compared with vehicle control group, IR induced distinguishable structural, while the treatment with WOPs showed little or no change in structure on both 3 and 14 days postirradiation (Figure 6). In addition, IR exhibited shortened villus height and increased crypt depth, which reversed in WOPs groups at both of the time points (Figure 7). Thus, WOPs mitigated mucosal injury especially in WOPs 0.44 g/kg group, which displayed more sound effect than whey protein at both of the time points. 

### 2.7. Effect of WOPs on Intestinal Permeability in Irradiated Mice 

**Figure 8:**
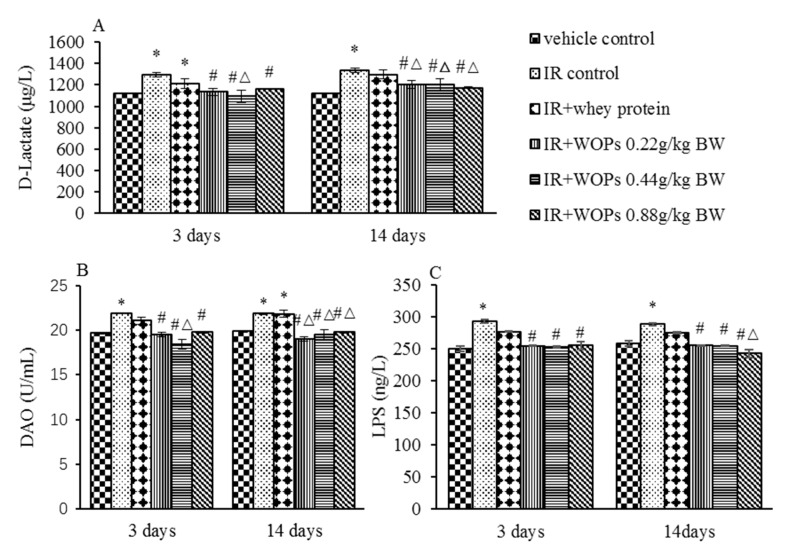
Effect of WOPs on intestinal permeability in irradiated mice. (**A**) Serum D-lactate content; (**B**) Serum DAO content; (**C**) Serum LPS content. Values represented the mean ± SD (*n* = 12 per group). * *p* < 0.05 versus vehicle control group, # *p* < 0.05 versus IR control, and Δ*p* < 0.05 versus IR+whey protein control group.

Compared with vehicle control group at 3 days and 14 days after irradiation IR induced the increased d-Lactate, DAO and LPS level in serum (Figure 8). In the WOPs groups, a significant decrease was noted in serum d-Lactate, DAO, and LPS level compared with IR control group at both time point. The treatment with 0.44 mg/kg BW WOPs had a more decreased level of d-Lactate and DAO than whey protein on the 3 days postirradiation, and the effect of other dose of WOPs was superior in a long term. However, the difference in serum LPS level between WOPs and whey protein was only significant on the 14 days after irradiation in 0.88 mg/kg BW dose.

### 2.8. Effect of WOPs on the Expression of Spleen Apoptosis Related Proteins

**Figure 9:**
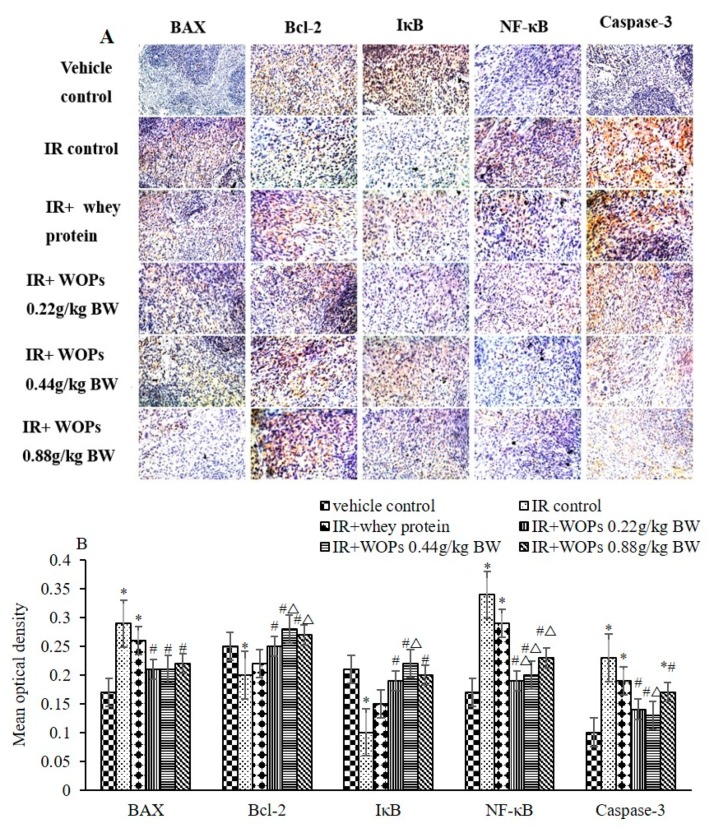
Effect of WOPs on the expression of spleen apoptosis related proteins. (**A**) The expression of BAX, Bcl-2, IB NF-B and Caspase-3; (**B**) mean optical density of BAX, Bcl-2, IB NF-B, and Caspase-3. Values represented the mean ± S.D. (*n* = 5 per group), which were analyzed by ANOVA test followed by least significant difference for post hoc test between multiple groups. * *p* < 0.05 versus vehicle control group, # *p* < 0.05 versus IR control, and Δ*p* < 0.05 versus IR+whey protein control group.

Figure 9 showed that the expression of pro-apoptotic proteins, such as Bax, NF-κB and caspase-3 were increased, the expression of anti-apoptotic protein Bcl-2 and IκB were decreased during the IR induced acute tissue damage. While 14-day pretreatment of WOPs suppressed the expression of pro-apoptotic proteins and promoted the expression of anti-apoptosis protein. The treatment of 0.44 mg/kg BW WOPs had a more decreased level expression of NF-κB and Caspase-3 and the increased level expression of Bcl-2 and IκB than the whey protein group.

## 3. Discussion

IR-induced damage is primarily attributed to free radicals, which play a major role in radiation effects on biological tissues and organisms [11]. Although the free radicals’ spectra produced by IR are same as those produced during metabolism, there are differences in their micro-distributions. Therefore, ROS produced under physiological conditions is an important signal molecule to regulate the biochemical process of cells, while excessive free radicals produced by IR has the harmful effect [12]. It has been reported that 90% of the IR- induced DNA damage is caused by free radicals [13]. Nevertheless, organisms have antioxidant defense systems for scavenging free radicals, including enzymatic and non-enzymatic antioxidant defense mechanisms, i.e. low molecular weight of antioxidant glutathione (GSH) and superoxide dismutase (SOD), glutathione peroxidase (GSH-Px), catalase (CAT) etc. GSH is the substrate or cofactor of antioxidant enzymes. SOD can transform O_2_^−^ into H_2_O_2_ by means of disproportionation. GSH-Px and CAT converted the latter into H_2_O and O_2_ [14,15]. During planned whole-body or partial-body radiation exposure scenarios, the body was so overwhelmed with free radicals that antioxidant defense systems were incompetent to effectively scavenge them. Antioxidants are known to scavenge free radicals, and therefore considered strong candidates for development of radioprotector [16]. The antioxidant capacities of bioactive peptides, in which different amino acid compositions have different validities, have been extensively studied [8,17,18]. Previous studies demonstrated that walnut protein hydrolysate was an effective antioxidant for scavenging various free radicals through different scavenging ways and make it a potential therapeutic agent for alleviation of memory impairment [19,20]. These studies prompted us to explore the efficiency of WOPs in the protection against radiation-induced damage.

Here we have used irradiated BALB/c mice as a damage model to the ^60^Co γ radiation and discovered the role of WOPs in scavenging free radicals and protecting radiosensitive tissue. We orally administered the mice with WOPs via drinking water, considering the fact that this administration route is more similar to the way human exposure to WOPs, while intragastric administration has certain injuries to mice, and it is difficult to eliminate bias caused by the different proficiency level of the operators. In order to determine the concentration of WOPs solution, we continuously monitored the water consumption of mice within 24 hours before the formal experiment. To guarantee that WOPs were effectively absorbed, we also monitored the water consumption daily during the experiment, and adjusted the concentration of WOPs in time. We had carefully prepared the solution to ensure that the WOPs were completely dissolved, thereby ensuring the efficient absorption. Previous studies in our lab also had used this route of administration and got fine results [21]. 

Specifically, our results have shown that ^60^Co γ radiation led to a robust increase in free radicals and the suppression in hematopoietic system, immune system, as well as the dysfunction of intestinal barrier. We found that the administration of WOPs augmented antioxidant defense system compared with non-administrated control mice, and there was significant trend toward higher survival rate and less weight loss in mice administrated with WOPs. Our data also suggested that WOPs accelerated hematopoietic recovery. The hematopoietic system is highly sensitive to IR and the doses of beyond 2 Gy can lead to myelosuppression like neutropenia, lymphocytopenia and thrombocytopenia, which therefore increases the risk of infection, bleeding, and even causes death. However, hematopoietic reconstitution relies solely on the self-renewal capability of hematopoietic stem progenitor cells, whereas antioxidants could improve its survival [22]. 

Radiation-induced apoptosis can be readily observed not only in proliferating stem cells such as bone marrow and jejuna crypt cells but also in splenocytes such as lymphocytes and granulocytes [23]. Previous studies stated that IR could result in a massive killing of blood cells such as lymphocytes and even in a halting of the proliferation of hematopoietic progenitors, thereby leading to the suppression of immune function [24,25]. Along this line, the protection of radiosensitive immune organs including spleen and thymus is rational for recovering from the injury induced by whole-body exposures to IR. In this study, we observed that γ-ray irradiation indeed resulted in greatly reduced spleen and thymus index, and augmented the expression of pro-apoptotic proteins while abated the anti-apoptotic protein in spleen, and WOPs administration appeared to be important for limiting this damage. However, a deficit in immune system may ultimately result in inflammation by weakening intestinal barrier function [26]. The expression of pro-inflammatory cytokines was regulated by transcription factor Nuclear FactorκB (NF-κB). After the exposure to IR, NF-κB activation is initially triggered by ataxia telangiectasia mutated protein (ATM) which is activated by DNA double strand breaks, thereby controls the expression of more than 100 pro-inflammatory genes, including *TNF-α, IL-1β, IL-6, CXCL8*, cyclooxygenase (*COX*2), vascular cell adhesion molecule-1 (*VCAM*1), and intercellular adhesion molecule (*ICAM*1) [27,28,29]. Our data revealed that 3.5 Gy γ-ray radiation resulted in a sharp decrease of IL-6 and an increased TNF-α level whereas WOPs administration reversed the trend. This suggested that WOPs administration was important to limit IR-induced inflammatory cascade through reducing DNA double strand breaks, ultimately suppressing activation of NF-κB.

Because of highly proliferating epithelium cells, the small intestine is one of the most radiosensitive organs, whose essential function is absorbing water and nutrients, regulating ionic homeostasis, exerting anti-microorganisms barrier function and modulating immune response. Thus, the intestinal mucosa has to serve a complex barrier function, in which the epithelium is in charge of regulating the selective transport of water, ions and nutrients. Evidence obtained in both animal models and humans is accumulating in support of a role of alterations of intestinal barrier function in a vast series of conditions, including radiation. This in turn opens up the possibility of managing these diseases by reinforcing intestinal barrier function [26], like animal model of colitis to study the effects of bioactive peptides, in which they ameliorated inflammation [30,31]. Different radioprotectors have been proposed to protect IR-induced damage, in which alteration in intestinal barrier function is also considered to play a role [5,32,33]. In this regard, intestine histopathology was carried out and d-lactate, DAO, and LPS in serum were detected to determine the effect of WOPs on the integrity of the epithelial barrier. Given that intestinal epithelial integrity is paramount importance to maintain intestine barrier function, and d-lactate, DAO and LPS are important indicators of intestinal integrity and barrier function, therefore when the intestinal barrier is damaged, there is an increase of them in the blood. Finally, we found that engagement of WOPs might offer a strategy to reduce intestine epithelial barrier dysfunction and promote epithelial integrity during the acute tissue damage caused by IR and thus help to prevent the development of inflammation. 

Above all, pre and post-treatment of WOPs may help to ameliorate acute damage, which is induced by ionizing radiation and accelerate its recovery in mice. However, the fundamental mechanisms responsible for the radioprotective effect of WOPs have not yet been fully elucidated. Our experimental studies suggested that the mechanism may be related to its antioxidant activity, protection of immune organs and intestinal barrier, inhibition of apoptosis and reduction of intestinal permeability. Furthermore, As shown in Table 1, the contents of arginine, phenylalanine, tyrosine and leucine were higher in WOPs. Evidences show that arginine can promote intestinal cell proliferation [34,35], increase immune function [36,37], improve the intestinal morphology, such as villus height and crypt depth [35], and attenuate oxidative stress [38]. However, inadequate phenylalanine (Phe) intake associated with disturbed macrophage function, which play a crucial role in intestinal immune barrier [39,40]. Another data suggest that Phe could reduce oxidative damage and improve intestinal physical barrier by up-regulating tight junction protein occludin and ZO-1 [41,42]. In addition, data showed that Phe as a precursor of melanin, may be involved in inhibiting the production of inflammatory factors such as interleukin-1b (IL-1b), interleukin6 (IL-6) and TNF-a in human peripheral blood monocytes and the mechanism may associate with modulating the signaling activity of NF-κB [42,43]. Tyrosine is a precursor of thyroid hormone that can activate mammalian target of rapamycin (mTOR) in human fibroblasts. While mTOR reduced TNF-a production by inhibiting NF-κB activation [43,44,45,46]. Therefore, it may also be one of the mechanisms that WOPs could ameliorate IR induced acute damage and accelerate its recovery in mice. Considerably more work, hopefully, will be done in this area.

There were several limitations in our study. First, although our data demonstrated the radioprotective effect of WOPs, the role of WOPs in tissue damage of tumor patients after radiotherapy remains unclear, causal relationships remain to be formally proven. Second, testing the role of WOPs in IR induced immunosuppressive animal models may allow more general conclusions to be drawn about the relevance of immune system in IR induced tissue damage.

## 4. Materials and Methods

### 4.1. Chemicals 

Walnut oligopeptides were supplied by Beijing Tiantai Biotechnology Co., Ltd. (Beijing, China), the content of the WOPs sample with relative molecular weight <1000 Da (small molecule oligopeptides) was 86.5%, and that of free amino acids amounted to 2.98 g per 100 g. The detailed information has been shown in Table 1.

### 4.2. Animals and Treatments

Female BALB/c mice (18–22 g) were obtained from the Animal Service of Health Science Center, Peking University. The mice were housed at a constant temperature (25 ± 1 ℃) and humidity (50–60%) under a 12 h: 12 h light-dark cycle with free access to standard food (American Institute of Nutrition Rodent Diets-93G (AIN-93G diet) and water. The protocol was reviewed and approved by the Institutional Animal Care and Use Committee of Peking University, and all animals were treated according to the Principles of Laboratory Animal Care (NIH publication No. 85-23, revised 1985) and the guidelines of Peking University Animal Research Committee (Laboratory animal production license No.: SCXK(Jing)2016-0010; Laboratory animal use license No.: SYXK(Jing)2016-0041).

After one week of acclimatization, the mice were randomized into six groups (*n* = 30): vehicle control group, irradiation (IR) control group, IR+whey protein group (0.44 g/kg) and three IR + GOP groups (0.22, 0.44, and 0.88 g/kg, respectively). Then, each group was randomized into three subgroups (*n* = 10), the first subgroup was used for survival assays; the other two subgroups were used for other assays on the 3rd and 14th day after irradiation. The mice in control group were administered with distilled water, and the other mice were administered with the different concentrations of whey protein and WOPs solution, respectively, through drinking.

### 4.3. Irradiation Exposure

The resource of ^60^Co-gamma radiation facility was provided by the Radiation Center in Peking University, Beijing, China. On the 15th administration day, except vehicle control group, the first subgroup mice used for survival assays were radiated with 8 Gy at a dose rate of 1 Gy/min, and the other two subgroups mice were radiated with 3.5 Gy at a dose rate of 1 Gy/min.

### 4.4. Survival Assays

Survival of the mice radiated with 8 Gy was monitored continuously for 30 days after radiation.

### 4.5. White Blood Cells Count and DNA Concentration in Bone Marrow Cells 

On the 14th day, caudal vein WBC was counted using an automated hematology analyzer (Beckman Coulter Inc., Brea, CA, USA), as described previously [5] and on the 15th day the mice were radiated with 3.5 Gy at a dose rate of 1 Gy/min. At 3 and 14 days postirradiation, caudal vein WBC was counted again. On the third day postirradiation, blood samples of the mice were collected from ophthalmic venous plexus and sacrificed by cervical dislocation. The thighbones were removed, and bone marrow cells were derived and passed into a 2-mL syringe through an 11-gauge needle [47]. The DNA concentration in the suspension of bone marrow cells was measured at 260 nm by FlexStation 2 fluorescence microplate reader [5] (Molecular Devices, Sunnyvale, CA, USA).

### 4.6. Histopathology Analysis and Crypt and Villus Morphometry

At 3 and 14 days postirradiation, the intestine was collected and fixed after the mice were sacrificed. Duodenum, jejunum and ileum segments were dehydrated and embedded in paraffin to produce four cross sections per segment in each mouse. The slides were stained with hematoxylin and eosin for histopathology analysis by light microscopy. About 20–40 separate measures of villus length and crypt depth per mouse were obtained to calculate the mean length and depth per mouse [5].

### 4.7. Enzyme-linked Immunosorbent Assay

The liver, spleen, thymus were removed and weighed after the mice were sacrificed. Superoxide dismutase (SOD), glutathione peroxidase (GSH-Px) and malondialdehyde (MDA) in serum and liver, and interleukin-6 (IL-6), tumor necrosis factor-α (TNF-α), d-Lactate, diamine oxidase (DAO), and endotoxin (LPS) in serum were determined by the mouse enzyme-linked immunosorbent assay kits purchased from Andygene Co. (Richardson, TX, USA).

### 4.8. Immunohistochemistry

The paraffin-embedded spleen tissue sections were used for immunohistochemistry. The expressions of apoptosis—related proteins, including Bax, Bcl-2, caspase, NF-κB and IκB, were detected using rabbit anti-Bax (1:250, ABcam, Cambridge, MA, USA), anti-Bcl-2 (1:100, ABcam), anti-caspase (1:100, ABcam), anti-NF-κB (1:1000, ABcam), and rabbit anti-IκB antibodies (1:100, Abcam), respectively, and then the sections were immunostained with goat anti-rabbit IgG conjugated to horseradish peroxidase using a DAB kit (ZhongShan Goldenbridge, Beijing, China). Each immunohistochemistry tissue section was analyzed by Image-Pro Plus 6.0 (Media Cybernetics Inc., Bethesda, MD, USA) to calculate the mean of optical density.

### 4.9. Statistical Analysis

Statistical analyses were performed using the SPSS software version 24 (SPSS Inc., Chicago, IL, USA). Data were expressed as mean ± standard deviation (SD) and analyzed by one-way analysis of variance (ANOVA) test; the difference of parametric samples among groups multiple comparison of least significant difference (equal variances assumed) or Dunnett’s T3 test (equal variances not assumed) was used. Survival curves of mice were estimated with the Kaplan–Meier analysis, and differences between groups were compared with the two-sided log-rank test. *P* < 0.05 indicated a statistically significant difference.

## 5. Conclusions

In conclusion, we have shown that administration of WOPs may offer radioprotective effect to augment antioxidant defense system and ameliorate immunosuppression, promote epithelial integrity ultimately limiting inflammation during acute tissue damage caused by IR. The mechanism may be related to its antioxidant activity, protection of immune organs and intestinal barrier, inhibition of apoptosis and reduction of intestinal permeability. Besides, the mechanism also associated with the amino acids composition of WOPs.

## Figures and Tables

**Table 1:** The Amino Acids Composition of WOPs.

Amino Acid	Content (g/100g)	Amino Acid	Content (g/100g)
Aspartic Acid	0.085	Cystine	0.001
Glutamic Acid	0.124	Valine	0.069
Serine	0.112	Methionine	0.020
Histidine	0.016	Phenylalanine	0.477
Glycine	0.058	Isoleucine	0.041
Threonine	0.150	Leucine	0.279
Arginine	1.015	Lysine	0.053
Alanine	0.132	Proline	0.057
Tyrosine	0.298		
